# Relationship between Long Chain *n*-3 Polyunsaturated Fatty Acids and Autism Spectrum Disorder: Systematic Review and Meta-Analysis of Case-Control and Randomised Controlled Trials

**DOI:** 10.3390/nu9020155

**Published:** 2017-02-19

**Authors:** Hajar Mazahery, Welma Stonehouse, Maryam Delshad, Marlena C. Kruger, Cathryn A. Conlon, Kathryn L. Beck, Pamela R. von Hurst

**Affiliations:** 1 Massey Institute of Food Science and Technology, School of Food and Nutrition, Massey University, Auckland 0745, New Zealand; h.mazahery@massey.ac.nz (H.M.); delshad.maryam@yahoo.com (M.D.); c.conlon@massey.ac.nz (C.A.C.); k.l.beck@massey.ac.nz (K.L.B.); 2 Commonwealth Scientific Industrial Research Organisation (CSIRO) Food, Nutrition and Bioproducts, Adelaide SA 5000, Australia; welma.stonehouse@csiro.au; 3 Massey Institute of Food Science and Technology, School of Food and Nutrition, Massey University, Palmerston North 4410, New Zealand; m.c.kruger@massey.ac.nz

**Keywords:** meta-analysis, omega-3, long chain polyunsaturated fatty acids, concentration, intervention, autism, symptoms

## Abstract

Omega-3 long chain polyunsaturated fatty acid supplementation (*n*-3 LCPUFA) for treatment of Autism Spectrum Disorder (ASD) is popular. The results of previous systematic reviews and meta-analyses of *n*-3 LCPUFA supplementation on ASD outcomes were inconclusive. Two meta-analyses were conducted; meta-analysis 1 compared blood levels of LCPUFA and their ratios arachidonic acid (ARA) to docosahexaenoic acid (DHA), ARA to eicosapentaenoic acid (EPA), or total *n*-6 to total *n*-3 LCPUFA in ASD to those of typically developing individuals (with no neurodevelopmental disorders), and meta-analysis 2 compared the effects of *n*-3 LCPUFA supplementation to placebo on symptoms of ASD. Case-control studies and randomised controlled trials (RCTs) were identified searching electronic databases up to May, 2016. Mean differences were pooled and analysed using inverse variance models. Heterogeneity was assessed using *I*^2^ statistic. Fifteen case-control studies (*n* = 1193) were reviewed. Compared with typically developed, ASD populations had lower DHA (−2.14 [95% CI −3.22 to −1.07]; *p* < 0.0001; *I*^2^ = 97%), EPA (−0.72 [95% CI −1.25 to −0.18]; *p* = 0.008; *I*^2^ = 88%), and ARA (−0.83 [95% CI, −1.48 to −0.17]; *p* = 0.01; *I*^2^ = 96%) and higher total *n*-6 LCPUFA to *n*-3 LCPUFA ratio (0.42 [95% CI 0.06 to 0.78]; *p* = 0.02; *I*^2^ = 74%). Four RCTs were included in meta-analysis 2 (*n* = 107). Compared with placebo, *n*-3 LCPUFA improved social interaction (−1.96 [95% CI −3.5 to −0.34]; *p* = 0.02; *I*^2^ = 0) and repetitive and restricted interests and behaviours (−1.08 [95% CI −2.17 to −0.01]; *p* = 0.05; *I*^2^ = 0). Populations with ASD have lower *n*-3 LCPUFA status and *n*-3 LCPUFA supplementation can potentially improve some ASD symptoms. Further research with large sample size and adequate study duration is warranted to confirm the efficacy of *n*-3 LCPUFA.

## 1. Introduction

The prevalence of Autism Spectrum Disorder (ASD) has dramatically increased over the past few years. While previous prevalence studies of ASD identified less than 10 in 10,000 individuals [1], recent estimates suggest rates of 90 to 250 in 10,000 individuals [2,3,4,5]. ASD is a life-long neurodevelopment disorder that appears during the first years of life [6]. Depending on the child’s predominant symptomatology, children with ASD exhibit difficulties with expressing and understanding certain emotions, understanding others’ mood, expressive language, and maintaining normal eye contact, as well as preference for minimal changes to routine, restricted ways of using toys and isolated play, all of which make it difficult for individuals to establish relationships with others, to act in an appropriate way and to live independently [6]. In addition, children with ASD frequently experience behaviour problems and medical conditions, including inflammation, oxidative stress, and autoimmune disorders [7,8,9,10,11,12], and altered brain structure and function (in a subset of individuals) [13,14]. The rising ASD rates are ascribed, in part, to a complex interaction between multiple genes and environmental risk factors [15], among which omega-3 long chain polyunsaturated fatty acids (*n*-3 LCPUFAs) is a strong candidate. LCPUFAs and their metabolic products have been implicated in ASD via their roles in brain structure and function, neurotransmission, cell membrane structure and microdomain organisation, inflammation, immunity and oxidative stress [16,17,18,19,20].

Blood polyunsaturated fatty acids (plasma, serum, red blood cell (RBC), and whole blood) levels are considered reliable biomarkers of their status [21]. Abnormality in blood levels of *n*-3 LCPUFA has been reported in psychiatric disorders including, but not limited to, attention deficit hyperactivity disorder (ADHD) and ASD [22,23,24]. Explanations for such abnormalities have been suggested to be lower dietary intake of *n*-3 LCPUFAs, and disturbances in fatty acid metabolism and incorporation of these fatty acids into cellular membranes in autistic populations compared to healthy controls [24,25,26]. A smattering of reports indicate differences in *n*-3 LCPUFAs, *n*-6 LCPUFAs and/or *n*-6 to *n*-3 LCPUFA ratios between populations with autism and healthy controls [14,26], but a few also failed to show any differences [27,28]. The reason for such discrepancies is not well examined, and there have been no attempts to systematically compare these studies. Hence, systematic analysis and synthesis of the evidence are warranted to determine if there are any differences in these blood fatty acids levels among healthy and individuals with ASD, and if so, whether *n*-3 LCPUFA supplementation may be beneficial in reducing symptoms in ASD.

To our knowledge, the efficacy of *n*-3 LCPUFA supplementation in ASD has been investigated by six open-label trials [29,30,31,32,33,34] and one case study [35], the majority of which (six out of seven studies) showed significant improvement in symptoms of ASD (Table S1). Despite this promising evidence, randomised controlled trials (RCTs) examining the beneficial effect of *n*-3 LCPUFAs in reducing symptoms of ASD have yielded inconclusive results. For example, Amminger et al. (2007) showed that supplementation with *n*-3 LCPUFA (EPA + DHA) was superior over placebo in reducing stereotypy, inappropriate speech and hyperactivity [36], while Mankad et al. (2015) failed to show any effect of *n*-3 LCPUFA supplementation on autism severity symptoms, adaptive functioning, externalizing behaviour or verbal ability [37].

To date, two systematic reviews of interventions with *n*-3 LCPUFA in ASD have been published [38,39]. In the review by Bent et al., published in 2009, authors set broad inclusion criteria and included all intervention trials of *n*-3 LCPUFAs of any type, dose, and duration addressing core and associated symptoms of ASD [38]. They identified six studies; one randomised controlled trial, four open-label trials and one case-study and concluded that the evidence was insufficient to support clinical recommendations [38].

Two years later, James, Montgomery and Williams (2011) published a Cochrane review including only two RCTs and performing meta-analyses on three primary outcomes (social interaction, communication and stereotypy) and one secondary outcome (hyperactivity) [39]. The authors reached the same conclusion as the Bent et al. review [38], and identified four ongoing studies. At the time of writing this review, the findings of one trial was published [37], one was terminated in 2014 (NCT01248130), and no information was available regarding the recruitment status or the availability of data for two trials (NCT00467818 and NCT01260961).

An updated systematic review is timely; more studies are now available, the prevalence of ASD is increasing together with a greater interest in the medical community (health professionals) on the beneficial effect of *n*-3 LCPUFA in the treatment of neurodevelopment disorders, as well as an increasing interest in using complementary and alternative medication in this population [40]. We aimed to conduct a current examination of evidence. We designed two systematic reviews and meta-analyses;

- Meta-analysis 1: a meta-analysis of evidence regarding blood *n*-3 LCPUFA levels in populations with ASD compared to typically developing counterparts (with no neurodevelopmental disorders) of any age and sex. A secondary aim for meta-analysis 1 was to perform a priori subgroup analysis to investigate the influence of ASD on fatty acid composition across different age groups (studies including only young children vs. studies also including children, teenagers, and adults).
- Meta-analysis 2: a meta-analysis of randomised controlled trials of *n*-3 LCPUFA supplementation (of any type, dose and duration) in ASD populations (of any age and sex) to assess the clinical efficacy of *n*-3 LCPUFAs treatment in reducing core symptoms of ASD and co-existing conditions.

## 2. Materials and Methods

All study procedures for both meta-analyses were pre-defined, but have not been registered or published elsewhere.

### 2.1. Eligibility Criteria

For meta-analysis 1, we included case-control observational studies that examined the differences in blood fatty acid levels between populations with ASD and healthy typically developing controls (with no neurodevelopmental disorders) of any age and sex. Studies were excluded it they included non-typically developing controls, were non-English or unpublished. Because DHA, EPA and ARA are amongst the most reported fatty acids of *n*-3 LCPUFAs and *n*-6 LCPUFAs categories, respectively, and have been shown to be more biologically active in the brain and been linked to neurodevelopment disorders, we focused on these fatty acids as well as the ratio of ARA to EPA and DHA and the ratio of *n*-6 LCPUFA to *n*-3 LCPUFA [41,42]. We included studies that reported LCPUFA in various blood fractions expressed as either % of total fatty acids or in concentration units, including RBC, serum, plasma, plasma phospholipids and whole blood [21]. These fractions have been shown to be reliable markers for the general fatty acid pool [21].

For meta-analysis 2, we included RCTs of any dose, type, and duration of *n*-3 LCPUFAs in participants with ASD of any age and sex who were randomised to receive either intervention or placebo, and reporting one of the following outcome measures: core symptoms of ASD including social interaction, communication, and repetitive restrictive behaviours or interests (RRB), and symptoms or behaviours associated with ASD including hyperactivity, irritability, sensory issues, and gastrointestinal symptoms. Unpublished and non-English studies were excluded.

Meta-analyses were performed if at least two studies employed the same assessment tool to measure the outcome of interest. There is a large variability in outcome assessment methods in ASD studies [43]. This use of different tools not only compromises the validity of a study by increasing the likelihood of type 1 error [44], but also complicates an effective comparison across studies.

### 2.2. Search Methods for Identification of Studies

We searched PubMed, MEDLINE, Web of Science, CINAHL, PsycINFO, PsycARTICLES and PsycNET up to May, 2016 to identify relevant studies in English. We employed broad search terms to include all potential studies that may fall within each of the mentioned reviews. The search strategy used the following terms: (“omega 3” OR “omega3” OR “omega-3” OR “polyunsaturated fatty acids” OR “polyunsaturated fatty acid” OR “essential fatty acids” OR “essential fatty acid”) AND (“autism” OR “autistic” OR “autism spectrum disorder” OR “Asperger”). We also reviewed the reference lists of all identified studies to identify additional studies. Results from each database were downloaded into EndNote (version X6, 2012, Thomson Reuters, Philadelphia, PA, USA). Duplicates were removed and abstracts were screened. When an abstract met the eligibility requirements, it was assigned to one of two meta-analyses and the full article was read to ensure the inclusion and exclusion criteria were met. The study identification was done by one investigator (H.M.).

### 2.3. Data Extraction, Management, and Quality Assessment

Two reviewers (H.M. and M.D.) independently performed data extraction from each study into pre-piloted extraction tables. Discrepancies in the data extraction were resolved by discussion and reaching consensus.

The following data were extracted for both meta-analyses: author, date of publication and setting, sources of funding, conflict of interest, aims, objectives and hypothesis, and population characteristics while extractions specific to each meta-analyses are described below.

For meta-analysis 1, the following data were also extracted: the mean and SD for blood *n*-3 LCPUFAs (DHA, EPA or total), *n*-6 LCPUFAs (ARA or total), and for *n*-6 to *n*-3 LCPUFA ratios (ARA to DHA, ARA to EPA, or total *n*-6 to total *n*-3 LCPUFA), fatty acid analysis method, the body tissue in which the fatty acid was measured, the unit of measure, and the significance value. If a study reported LCPUFA in two different blood tissues, the priority was given to RBC, followed by plasma phospholipids, serum/plasma, and whole blood. While RBC and plasma phospholipids LCPUFA reflects long-term fatty acid intake, serum/plasma or whole blood LCPUFA are influenced by recent intake of these fatty acids [21,45]. If a study reported both relative and absolute measures, the former measures were included in the meta-analysis to limit the methodological heterogeneity. The method by which blood fatty acid composition is expressed (relative vs. absolute) has been shown to modify the LCPUFA—disease relationship [46]. Inter-study variation in extraction and separation efficiencies in fatty acid analyses can be overcome by relative expression of fatty acids (expressed as a percentage of a fatty acid normalised to the total amount of all measured fatty acids in a sample) [46]. If more than two groups were included, only relevant groups were selected. A quality appraisal was performed in duplicate by two investigators (H.M. and M.D.) using the “Health Canada Quality Appraisal Tools for Observational Studies” [47]. A quality score of ≤6 was considered lower quality [47]. No studies were excluded based on quality scores, but sensitivity analysis was performed to assess the impact of these studies on the overall results.

For meta-analysis 2, study design, intervention (the dose of intervention was converted, where required, to gram from milligram for easy comparison), delivery method, compliance, intervention period, outcome measures, assessment tools, results, conclusion, potential confounders and assessment of bias risk following Cochrane bias risk assessment including selection, performance, detection, attrition, reporting and commercial bias were also extracted [48]. A further quality appraisal was performed in duplicate by two investigators (H.M. and M.D.) using the “Health Canada Quality Appraisal Tools for Randomised Controlled Trials” to assess the quality of the individual studies [47]. A quality score of ≤7 was considered lower quality [47].

### 2.4. Statistical Analysis

Both meta-analyses were performed using Review Manager (RevMan, version 5.3, The Nordic Cochrane Centre, Copenhagen, Denmark).

#### 2.4.1. Meta-Analysis 1

For each outcome, the mean and SD for each study group (cases and controls) was entered into Review Manager. If *n*-3 to *n*-6 fatty acid (total *n*-3 to *n*-6 LCPUFA, EPA to ARA or DHA to ARA) was reported, the ratio was converted to *n*-6/*n*-3 (1/ratio). The Review Manager calculator (between group differences) was employed to calculate the SD for these reverse ratios. The reverse ratio and SD were calculated for five studies [49,50,51,52,53].

The primary meta-analysis compared mean differences (95% confidence intervals (CI)) in outcomes across study groups. Due to significant heterogeneity a random effects model was used to calculate the forest plots with standardised mean differences and 95% CI. Standardised mean differences were calculated because blood levels of LCPUFA were measured and reported in different ways. A combination of Chi^2^-statistic (*p* < 0.1), *I*^2^ statistics (*I*^2^ 0%–40%, low; 30%–60%, moderate; 50%–90%, substantial; 75%–100%, considerable heterogeneity), and considering the variation of point estimates and the overlap of CIs across different studies was performed to measure heterogeneity [48].

To avoid false positive or negative results, we limited the number of subgroup analyses to one (stratified by age) and sensitivity analyses to three (blood tissue type, study quality, and author’s calculations). Then a priori subgroup analysis was performed using Chi^2^-statistic with a *p* value of <0.05 taken to indicate statistical significance [48]. We could not conduct meta-regression to investigate the impact of the potential mediators (location, sex, and the way by which fatty acid composition is expressed) due to the limited study numbers. To include one mediator in the analysis, at least 10 studies are required [48]. These variables were however carefully examined when the results were interpreted. Publication bias was examined using funnel plots in which the SE of the studies were plotted against their corresponding effect sizes.

#### 2.4.2. Meta-Analysis 2

For each outcome, the mean change and SD of change from baseline to endpoint for each intervention group (*n*-3 LCPUFA and placebo) was entered into Review Manager. If only baseline and end data were available the mean change was calculated by deducting the baseline from the end value, and the SD was then imputed from a mean correlation coefficient for an outcome from other studies in the meta-analysis. Standard deviations were calculated for one study [54]. Study authors were contacted for missing data, and if no response was received the data was not included in the meta-analysis. Data was unable to be retrieved for three studies [55,56,57].

The primary meta-analyses compared mean (95% CI) differences (net change in scores) in each domain between *n*-3 LCPUFA and control groups. Heterogeneity between studies was small hence a fixed-effects model was used to calculate forest plots with mean differences and 95% CI. Heterogeneity between studies was indicated using the same analyses employed in meta-analysis 1. No subgroup analyses or meta-regression were performed due to limited number of studies included in the meta-analysis. However, one sensitivity analysis was performed to evaluate the impact of calculations (SDs) and major methodological differences on heterogeneity and the overall results. Publication bias was not assessed due to the small number of studies included in this meta-analysis.

## 3. Results

**Figure 1:**
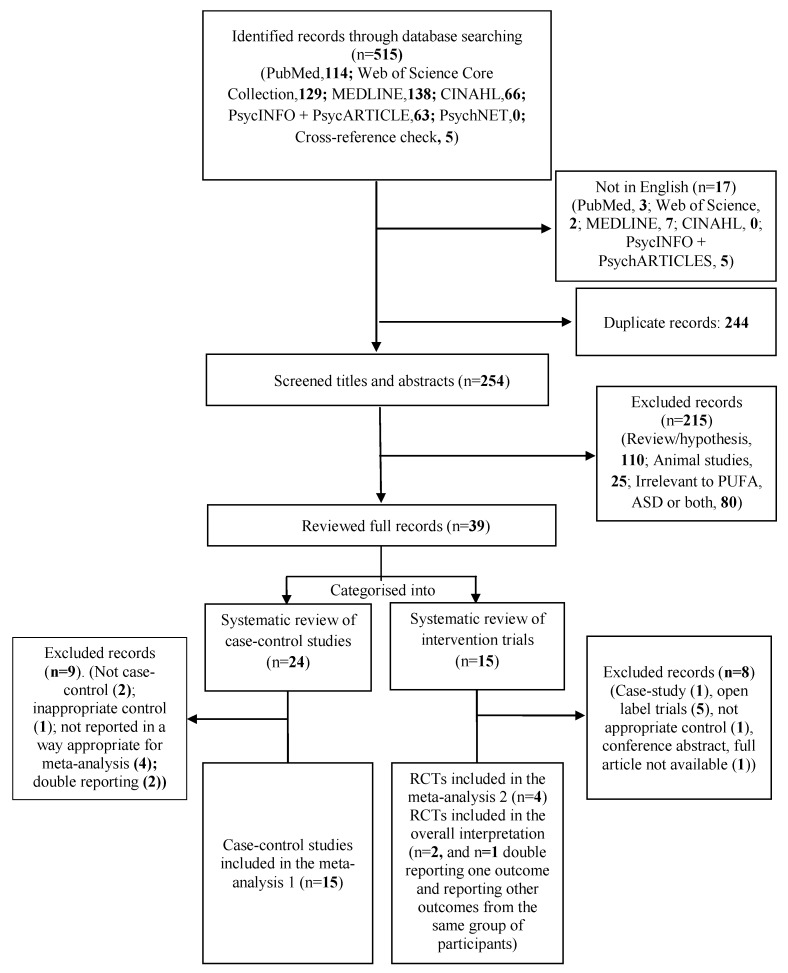
Flow diagram for selection of studies (PRISMA flow diagram).

From the initial searches, 510 articles and from the cross-reference check, five articles were retrieved. Titles and abstracts of 254 articles were screened after non-English and duplicates were removed. At this level, 216 were excluded as not relevant to the current topic. The remaining 38 articles were read and categorised into two groups; 24 articles into the case-control studies group and 15 into the intervention group (Figure 1, PRISMA Flow Diagram).

### 3.1. Systematic Review and Meta-Analysis 1

Of the 24 articles identified for meta-analysis 1, 15 were included in the meta-analysis. Reasons for exclusion were: not a case-control design [58,59], inappropriate control [25], the data not reported in a form suitable for analysis [26,60,61,62], and double-reporting [63,64]. Characteristics of included studies can be found in Table 1.

The majority of studies were conducted in the Middle East (*n* = 5; 2 Saudi Arabia (from one study group), 1 Oman, 2 Egypt) and Europe (*n* = 4; 2 UK, 1 Belgium, 1 Italy), with others conducted in the US (*n* = 2), Latin America (*n* = 1), Canada (*n* = 1), Asia (Japan, *n* = 1), and Australia (*n* = 1).

The 15 studies included 623 children and young people with ASD and 570 controls. Most studies included children under the age of 12, while a few included teenagers and adults also (*n* = 3) [24,49,53]. One study included adults up to age 22 years [49]. Cases and controls were matched on both age and sex (*n* = 8), two of which included other attributes such as IQ, home environment and dietary intake (*n* = 1) or geographical region (*n* = 1). Others matched two groups on either age (*n* = 3) or sex (*n* = 1), and one study included only males. Matching of cases and controls was not reported in two studies. In those studies including both sexes and reporting the sex distribution, the male/female ratio ranged from 2/1 to 12/1.

Most studies did not report the fasting state of blood samples while one study analysed non-fasting blood samples, and five studies fasting blood sample (ranging from 2 h to overnight fasting) [28,49,50,51,65]. Fasting state is considered to affect fatty acid composition measured in plasma/serum but not in RBC [21,45]. While most studies reported serum/plasma fatty acid composition, four studies reported RBC levels [24,28,52,66] and two reported both [27,51]. Most studies reported relative levels while five studies reported absolute levels (all from the Middle East) [26,30,50,65,67], and one both levels [53]. Sensitivity analysis showed no impact of blood tissue type and the way by which fatty acid composition is expressed on the heterogeneity. However, the way by which fatty acid composition is expressed affected the overall effect size for some measures (Refer to the next section).

The majority of studies reported DHA, EPA and ARA levels while two studies did not report levels of EPA [26,67], and one ARA [67]. Five studies reported both total *n*-3 LCPUFA and total *n*-6 LCPUFA levels. One study did not report either of the mentioned measures but the ratio of ARA to DHA and ARA to EPA [50]. Total *n*-6/*n*-3 LCPUFA ratio was reported in six studies, of which two reported ARA/EPA ratio [27,52], and one reported both ARA/EPA and ARA/DHA also [51]. Of the remaining studies, two reported the ratio of ARA/EPA [28,66] and ARA/DHA each [26,30], one both [53], and three no ratios [65,67,68]. Reverse ratios and SDs were calculated in five studies ([50] (only the ARA to EPA) and [49,51,52,53]). With the exception of one study [50] (refer to the next section), sensitivity analysis showed no impact of calculation on the overall results.

All studies included in the review scored between four and nine points out of a possible 11 in our quality assessment tool, with three studies scoring ≤ 6 [24,66,67] (Table S2). It should be noted that the maximum score for the “Health Canada Quality Appraisal Tools for Observational Studies” is 12 [47] but because “measuring the exposure in duplicate or more” is of no relevance for case-control studies and all studies received a score of “0” for this criterion, the maximum score adds up to 11 for this review. Studies with scores of ≥7 are considered having higher quality. Sensitivity analysis showed no impact of removing studies with a quality score ≤6 on the heterogeneity or overall results.

The quality criteria failed by most studies were attrition and the reasons for attrition. Although attrition is not important for case-control studies, it could be of relevance to ASD clinical studies. Drop out of some populations with particular characteristics such as high anxiety levels or sensory issues (a distinct criterion under RRB domain in ASD diagnosis [6]) that are associated with fear of blood test and consequently inability to obtain a blood sample or withdrawal from a study could affect the external validity of a study. The most prevalent confounding factors that were not controlled for in statistical analysis were dietary intake of LCPUFA and medication use. No conflict of interest that may have affected study outcomes was apparent in any study.

#### 3.1.1. Individual *n*-3 LCPUFA (DHA, EPA, and ARA) and Their Ratios (ARA to DHA and ARA to EPA)

**Figure 2:**
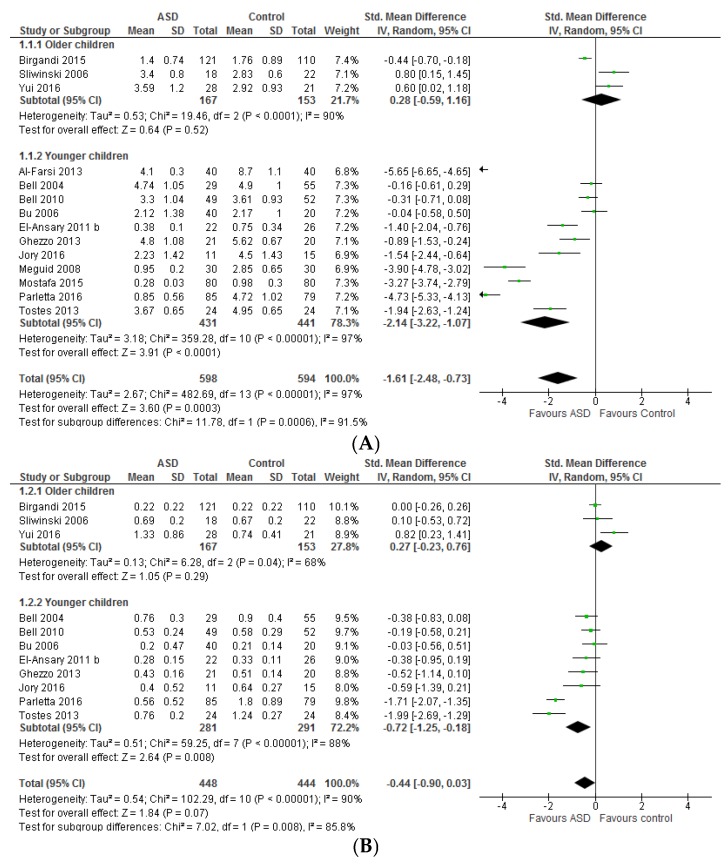

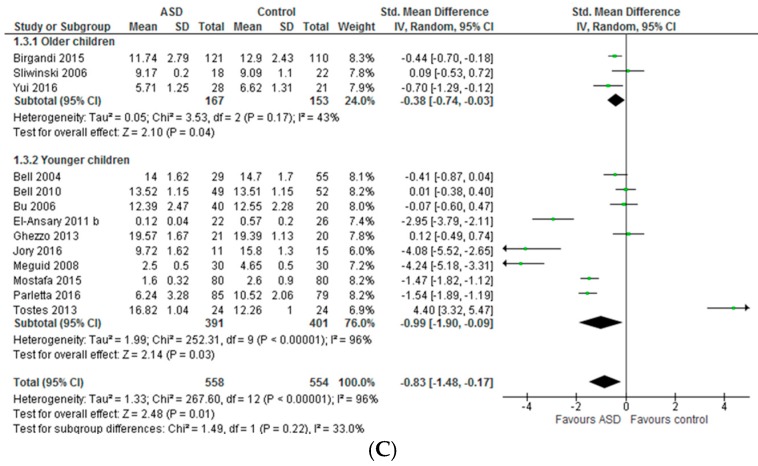
Forest plots of mean (95% confidence interval (CI)) weighted difference in blood levels of docosahexaenoic acid (DHA) (**A**); eicosapentaenoic acid (EPA) (**B**); and arachidonic acid (ARA) (**C**) between populations with Autism Spectrum Disorder (ASD) and typically developing controls stratified for subgroups with studies including all age groups (children, teenagers, and adults) vs. those including children only. Direction of effect (negative, lower mean in ASD group; positive, lower mean in control group; zero, no difference between groups).

Significant differences were seen between those studies that recruited children only vs. those that also included teenagers and adults for blood levels of DHA and EPA (Chi^2^ = 11.78, *p* = 0.0006 and Chi^2^ = 7.02, *p* = 0.008, respectively) but not ARA (Chi^2^ = 1.49, *p* = 0.22) (Figure 2). Hence results for DHA and EPA for these subgroups were described separately. For ARA, the results described are from all studies combined.

Overall, in the younger age group studies, ASD children had significantly lower DHA and EPA levels than typically developing controls (standardised mean difference (95% CI) −2.14 [−3.22, −1.07], *Z* = 3.91, *p* < 0.0001 and −0.72 [−1.25, −0.18], *Z* = 2.64, *p* = 0.008, respectively). Considerable heterogeneity was seen for DHA (*I*^2^ = 97%, *p* < 0.00001) and EPA (*I*^2^ = 88%, *p* < 0.00001). Heterogeneity for DHA was not altered by removing any studies. Heterogeneity for EPA reduced slightly by removing the Parletta, 2016 study [52] (*I*^2^ = 74%, *p* = 0.0008). Removal of this study together with the Tostes, 2013 study [68] significantly reduced heterogeneity for EPA (*I*^2^ = 0%, *p* = 0.78) that was accompanied by a reduction in the difference between cases and controls (−0.30 [−0.51, −0.08], *Z* = 2.73, *p* = 0.006, *n* = 356). These two studies were different with respect to some characteristics that may affect outcomes compared to other studies; children with ASD were significantly younger than typically developing children in the Parletta, 2016 study [52], and 88% of children with ASD in the Tostes, 2013 study were on psychotropic drugs [68].

In studies including all age groups (children, teenagers, and adults), no significant differences were seen in DHA and EPA levels between cases and controls (0.28 [−0.59, 1.16] and 0.27 [−0.23, 0.76], respectively). Heterogeneity for DHA and EPA was substantial (*I*^2^ = 90%, *p* = 0.0001 and *I*^2^ = 68%, *p* = 0.04, respectively). Removal of the Brigandi, 2015 study [24] reduced the heterogeneity significantly for DHA (*I*^2^ = 0%, *p* = 0.65) and slightly for EPA (*I*^2^ = 64%, *p* = 0.10). Removal of this study resulted in children with ASD having significantly higher DHA (0.69 [0.26, 1.12], *Z* = 3.13, *p* = 0.002, *n* = 89) levels than typically developing controls but no impact on EPA. The Brigandi, 2015 study [24] was different from the other two studies in this subgroup in that both classic and regressive type ASD were included, cases and controls were not matched by any attributes, intellectual functioning of patients was not considered, and this study had a low quality appraisal score. The Sliwinski, 2006 [49] and Yui, 2016 [53] studies included ASD patients with a borderline or normal intellectual functioning (IQ > 70). The results should be interpreted with caution because the number of studies included is small (*n* = 3).

With regard to ARA, children with ASD had significantly lower ARA levels than typically developing controls (−0.83 [−1.48, −0.17], *Z* = 2.48, *p* = 0.01), but heterogeneity between studies was substantial (*I*^2^ = 96%, *p* = 0.00001). Heterogeneity was not reduced by excluding any single study. However, removing studies that reported absolute levels [26,30,65] resulted in a smaller overall effect estimate (−0.24 [−0.88, 0.41], *Z* = 0.71, *p* = 0.48, *n* = 840).

**Figure 3:**
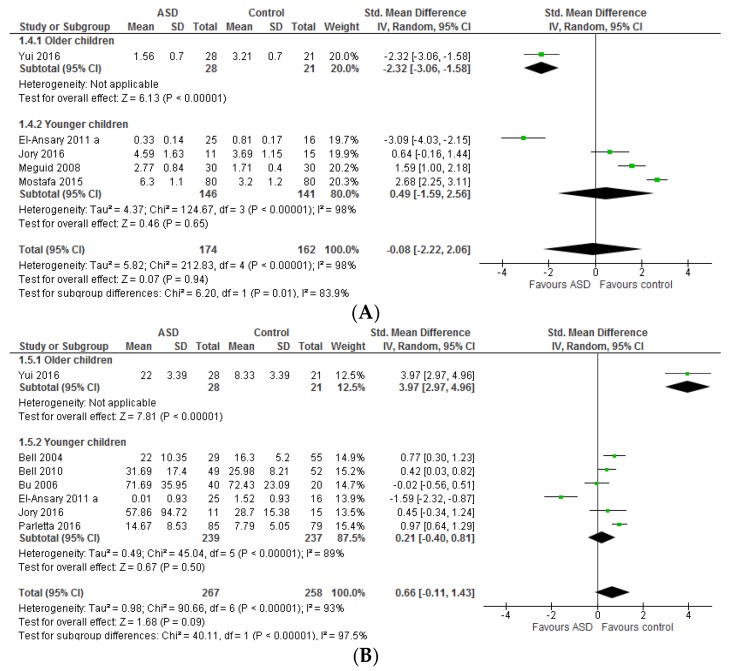
Forest plots of mean (95% confidence interval (CI)) weighted difference in the ratio of arachidonic acid (ARA) to docosahexaenoic acid (DHA) (**A**) and the ratio of ARA to eicosapentaenoic acid (EPA) (**B**) between populations with Autism Spectrum Disorder (ASD) and typically developing children stratified for subgroups with studies including all age groups (children, teenagers, and adults) vs. young children only. Direction of effect (negative, lower mean in ASD group; positive, lower mean in control group; zero, no difference between groups).

Only one study that included all age groups (children, teenagers, and adults) reported either ARA/DHA or ARA/EPA [53] thus the combined results of older and younger children are described (Figure 3). The ratio of ARA/DHA and ARA/EPA did not differ significantly between ASD populations and typically developing controls (*p* = 0.94 and *p* = 0.09, respectively). The heterogeneity was considerably high for both ratios. Heterogeneity was not reduced by excluding any studies (both ARA/DHA and ARA/EPA). With regard to ARA/EPA, however, the overall effect estimate changed considerably by the removal of El-Ansary, 2011a study [50] (0.99 [0.32, 1.67], *Z* = 2.88, *p* = 0.004, *I*^2^ = 91%). Cases and controls were not matched on sex in this study, the reverse ratio and SD was calculated, and the absolute level was reported.

#### 3.1.2. Total *n*-3 and *n*-6 LCPUFA and Their Ratios

**Figure 4:**
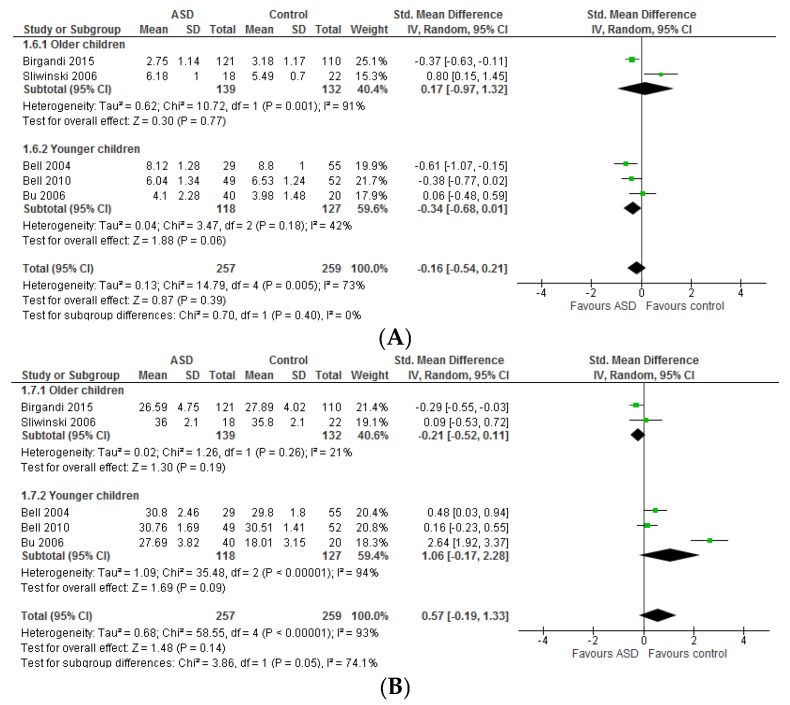
Forest plots of mean (95% confidence interval (CI)) weighted difference in the total *n*-3 long chain polyunsaturated fatty acids (*n*-3 LCPUFA) (**A**) and total *n*-6 long chain polyunsaturated fatty acids (*n*-6 LCPUFA) (**B**) between populations with Autism Spectrum Disorder (ASD) and typically developing children stratified for subgroups with studies including all age groups (children, teenagers, and adults) vs. young children only. Direction of effect (negative, lower mean in ASD group; positive, lower mean in control group; zero, no difference between groups).

No significant differences were observed in total *n*-3 and *n*-6 LCPUFA between studies including young children only and those including all age groups (children, teenagers, and adults) (Chi^2^ = 0.70, *p* = 0.40 and Chi^2^ = 3.84, *p* = 0.05, respectively) thus the results for the combined groups are described (Figure 4).

The pooled standard mean differences for the total *n*-3 LCPUFA and total *n*-6 LCPUFA between ASD and typically developing children were −0.16 [−0.54, 0.21] (*I*^2^ = 73%, substantial heterogeneity) and 0.57 [−0.19, 1.33] (*I*^2^ = 93%, considerable heterogeneity), respectively. Both were not statistically significant. Excluding comparisons that included teenagers and adults also [24,49] reduced heterogeneity in total *n*-3 LCPUA (*I*^2^ = 42%, *p* = 0.18) (−0.34 [−0.68, 0.01], *Z* = 1.88, *p* = 0.06, *n* = 245). Heterogeneity was reduced to an acceptable level when the Sliwinski, 2006 study [49] only was removed (*I*^2^ = 14%, *p* = 0.32) (−0.36 [−0.56, −0.15], *Z* = 3.40, *p* = 0.0007, *n* = 472). The Sliwinski, 2006 study [49] was different from other studies with respect to several characteristics; it included post pubertal youngsters up to age 22 years, it was a male-only study, and included those with an IQ > 55. Heterogeneity in total *n*-6 LCPUFA was not altered by the removal of any studies.

**Figure 5:**
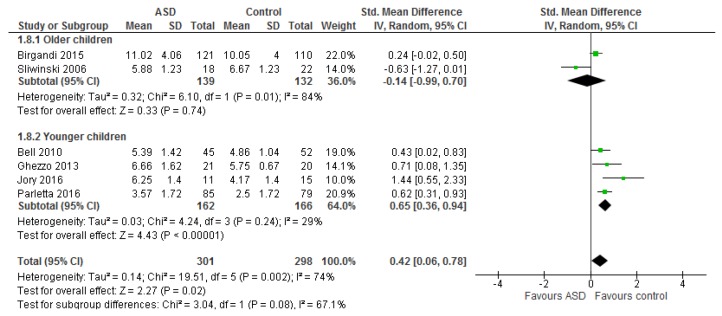
Forest plot of mean (95% confidence interval (CI)) weighted difference in the ratio of total *n*-6 long chain polyunsaturated fatty acids (*n*-6 LCPUFA) to total *n*-3 long chain polyunsaturated fatty acids (*n*-3 LCPUFA) between populations with Autism Spectrum Disorder (ASD) and typically developing children stratified for subgroups with studies including all age groups (children, teenagers, and adults) vs. young children only. Direction of effect (negative, lower mean in ASD group; positive, lower mean in control group; zero, no difference between groups).

The ratio of total *n*-6 LCPUFA to *n*-3 LCPUFA did not differ significantly between studies including young children only and those including all age groups (children, teenagers, and adults) (Chi^2^ = 3.04, *p* = 0.08) thus the overall results are described here (Figure 5). Children with ASD had a significantly higher *n*-6 LCPUFA to *n*-3 LCPUFA ratio (0.42 [0.06, 0.78], *Z* = 2.27, *p* = 0.02). The heterogeneity was substantial and was decreased by the exclusion of comparisons that also included teenagers and adults [24,49] (*I*^2^ = 29%, *p* = 0.24). The difference between cases and controls as well as the effect size increased considerably, 0.65 [0.36, 0.94], *Z* = 4.43, *p* < 0.00001, *n* = 328.

The funnel plots for DHA and ARA, indicated publication bias with a lack of smaller studies [studies with larger standard errors (SEs)] reporting negative results. Examination of funnel plot for EPA indicated no evidence of publication bias.

### 3.2. Systematic Review and Meta-Analysis 2

Of the 15 RCTs identified for systematic review and meta-analysis 2, four were included in the meta-analysis [36,54,69,70], two were included in the overall interpretation but not included in the meta-analysis [37,55], one was a double-reporting of one outcome [54] and reporting other outcomes from the same group of participants [57], and eight were excluded (Figure 1). Reasons for not being included in the meta-analysis 2 but being included in the overall interpretation were: data not reported in a format suitable for analysis [55], and use of an assessment tool not used by others [37]. Reasons for complete exclusion were: a conference paper with unpublished results at the time of writing this review [56], one open label randomised parallel intervention trial including a low sugar healthy diet as the control [29], and not being a RCT (*n* = 6, one case-study and 5 open label trials [30,31,32,33,34,35]). Characteristics of included studies can be found in Table 2 and of excluded studies in Table S1.

Of those included in the meta-analysis 2, two were conducted in the US [69,70], one in Austria [36], and one in Japan [54]. In the four studies, 55 participants with ASD received *n*-3 LCPUFA supplements and 52 received placebo. The Bent, 2011 and 2014 studies included children under the age of 8 years [69,70], the Amminger, 2007 study included children under 17 years [36], and the Yui, 2011 study included children older than 6 years and adults up to 28 years [54].

Of the two studies that could not be included in the meta-analysis, one study was from the US [55] and one from Canada [37] together including 37 and 34 individuals in the intervention and placebo groups, respectively. Children under the age of 5 and 10 years were included in the Mankad, 2015 and Voigt, 2014 studies, respectively [37,55].

All but one (the Amminger, 2007 study included only males [36]) included both males and females. Study groups were not matched on sex in these trials. The male to female ratio ranged from 3/1 to 12/1.

Of the RCTs included in the meta-analysis 2, the severity of autism at baseline was not considered in two trials [36,70], and the other two included patients with pre-defined severity (moderate severity and ABC social withdrawal subscale of >10 in the Bent, 2011 and Yui, 2011 studies, respectively) and IQ level (>50 and >80 in the Bent, 2011 and Yui, 2011 studies, respectively [54,69]). Of those included in the overall interpretation, the Voigt, 2014 study included patients with pre-defined severity (CARS score of >30 [55] and the Mankad, 2015 study equally distributed the severity across groups [37]. Co-existing problem behaviour was an inclusion criteria in two trials entered the meta-analysis 2 (hyperactivity in the Amminger, 2007 [36] and Bent, 2014 studies [70]).

Study length ranged from 6–16 weeks in RCTs included in the meta-analysis 2 and was 26 weeks in RCTs included in the overall interpretation. The majority of participants were supplemented with both EPA and DHA. The Yui, study (2011 and 2012) [54,57] used a combination of DHA and AA and the Voigt, 2014 study used only DHA [55]. Intake of EPA and DHA ranged from 0.70 to 0.84 g/day and 0.24 to 0.70 g/day, respectively, with the Yui, 2011 (and 2012) and Voigt, 2014 studies having the lowest DHA dose [54,55,57]. The Mankad, 2015 study reported an initial total dose of EPA and DHA of 0.75 g/day for two weeks which was doubled when tolerability to that dose was determined [37]. Placebos used were olive oil [37,54,57], safflower oil [69,70], corn oil + soybean oil [55], and coconut oil [36].

Behaviours were assessed using a variety of assessment tools (ranging from one to six tools used in each study) including, Aberrant Behaviour Checklist (ABC) ([36,54,55,69,70] but the outcome was not reported), Social Responsiveness Scale (SRS) [57,69,70], Behaviour Assessment System for Children (BASC) [37,55,69], Clinical Global Impression (CGI) [37,55,69,70], and further several tools that were used in isolation. Of these, full data was available for only four studies using the same assessment tool (ABC) [36,54,69,70].

All studies included in the review were of good quality, scoring 11–14 points out of a possible 15 (Table S3). Studies with scores of >7 are considered as having higher quality.

The quality criteria failed by most studies were whether intention-to-treat or per-protocol analysis was conducted (though according to final sample size analysed—larger than the sample size with drop outs deducted—it was apparent that the majority employed intention-to-treat analysis), and controlling for potential confounders. The most prevalent potential confounding factors that were not reported or reported but not considered in statistical analysis were dietary intake of LCPUFA or baseline LCPUFA status (as a measure of LCPUFA status, either dietary intake or blood level of LCPUFA needs to be reported).With the exception of the Bent, 2011 study [69] which investigated the impact of baseline LCPUFA status on behavioural changes in response to supplementation, the majority of studies failed to examine such a relationship while assessing baseline LCPUFA status [37,54,55,57]. Further factors were compliance (not reported in most studies) and medical regimen (most studies recruited patients on a stable medical regimen but did not report the type of regimen and its distribution across groups). It is also worth noting that a small number of females were included in these studies (with a male/female ratio ranging from 1/3 to 1/12) which could be a limitation in terms of generalisability, though reflecting the gender distribution of ASD.

The risk of bias for each study is summarized in Figure S1. Participants in the Amminger, 2007 and Yui 2011 (and 2012) studies were reported to be randomised but no details were available for random sequence generation [36,54,57]. Other studies used computer generated number [69,70] and block randomisation stratified by attributes including severity [37] and sex [55]. With the exception of the Amminger, 2007 study [36], randomisation was prepared by a third party in all trials [37,54,55,57,69,70]. The Amminger, 2007 provided no details regarding who performed the randomization [36]. All studies were reported as double-blinded (both researchers/assessors and participants) [37,54,55,57,69,70]. However, in the Amminger, 2007 study it is unclear if the researchers/assessors were blinded [36]. It is also unclear when researchers/assessors and participants were unblinded in the Amminger, 2007, Yui, 2011 (and 2012), and Mankad, 2015 studies [36,37,54,57]. The blinding was kept for the entire study (including data analyses) in the Bent, 2011, Bent 2014, and Voigt, 2014 studies [55,69,70]. With the exception of the Yui, 2011 (and 2012, no drop outs) [54,57] and Bent, 2014 (all included in the final analyses) [70] studies, participants were lost to follow up in the Amminger, 2007 (one individual from control) [36], Mankad, 2015 (one individual from *n*-3 LCPUFA group) [37], Bent 2011 (two individuals; one from each arm) [69], and Voigt, 2014 (five from *n*-3 LCPUFA and nine from control) [55] studies. The reason for drop outs in the Voigt, 2014 study were difficulty with participation (four from *n*-3 LCPUFA and three from control), trouble taking the supplements (one from *n*-3 LCPUFA and three from control), and concerns about supplement side effects (three from control) [55]. Regarding the latter concern, it is not clear if participants withdrew due to worsening behaviour, not observing any improvement, or because of an actual side effect during the intervention. With the exception of the Voigt, 2014 study [55], all outcomes in all trials were reported. The Voigt, 2014 study examined 52 behavioural subscales but only three outcomes were reported [55]. It is worth noting that the primary outcome was the measure of CGI which was completely reported. Other sources of bias including commercial bias were not apparent in any study.

#### 3.2.1. Effect of *n*-3 LCPUFA on Core Symptoms of ASD

**Figure 6:**
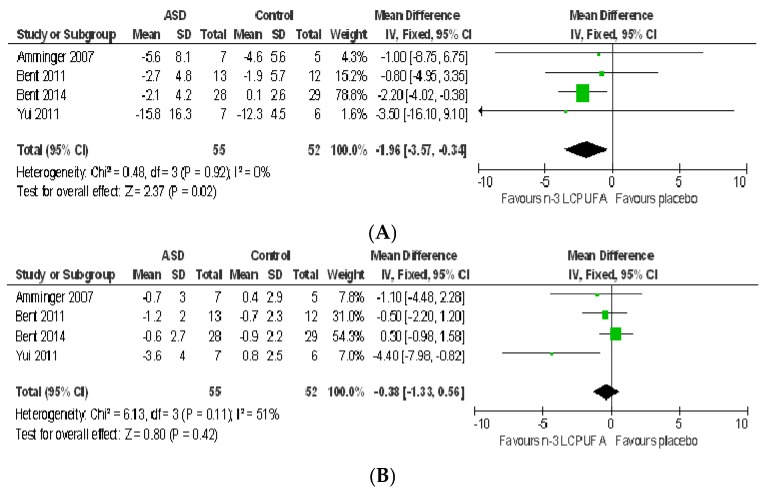

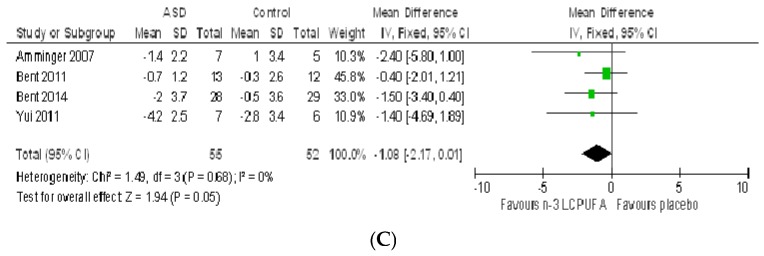
Forest plot of mean (95% confidence interval (CI)) fixed difference in change in social interaction (ABC) (**A**); communication (ABC) (**B**); and repetitive and restricted interests and behaviours (ABC) (**C**) in populations with Autism Spectrum Disorder (ASD) receiving *n*-3 long chain polyunsaturated fatty acid supplementation (LCPUFA) and placebo. Direction of effect (negative, more improvement in *n*-3 LCPUFA groups; positive, more improvement in placebo group; zero, no difference between groups).

**Social interaction:** The fixed mean difference for social interaction (assessed using ABC) significantly favoured *n*-3 LCPUFA with small effect (−1.96 [−3.5, −0.34], *Z* = 2.37, *p* = 0.02) and no heterogeneity (*I*^2^ = 0%, *p* = 0.92) (Figure 6A). Removing the Yui, 2011 study [54] did not change the results. The Yui, 2011 study [54] differs from others in that their sample included older participants (2–28 years) and those with IQ > 50, the daily dose of DHA was lower (0.24 g/day) and ARA (0.24 g/day) was added to the supplement, they used different dosing regimens for different age groups (half a dose for children aged 6–10 years) and SD was imputed resulting in a substantially greater SD in the *n*-3 LCPUFA group compared to the placebo group and other studies. Using SRS social interaction sub-domains (social motivation, social cognition, and social awareness), the Bent, 2014 study [70] found no effect of *n*-3 LCPUFA on social interaction (all domains > 0.05). Similarly, the Yui, 2012 study [57] did not find any effect of *n*-3 LCPUFA on any sub-domains of social interaction (measured by SRS, all *p* > 0.05). The social interaction in response to intervention did not differ across groups in the Mankad, 2015 study [37] where the authors used other assessment tools. The mean change scores decreased (showing an improvement) in both treatment groups in these studies. Voigt et al. (2014) found a significant difference in BASC social skills, favouring *n*-3 LCPUFA (−0.2 vs. 3.0, *p* = 0.04), which disappeared after correction for multiple comparisons [55].

**Communication:** Communication scores (assessed using ABC) did not differ between *n*-3 LCPUFA and placebo groups (−0.38 [−1.33, 0.56], *p* = 0.42) (Figure 6B). Moderate heterogeneity was seen in the meta-analysis for communication (*I*^2^ = 51%, *p* = 0.11). Removing the Yui, 2011 study [54] reduced the heterogeneity to 0% but had no impact on the overall result. Bent et al. (2011) and Bent et al. (2014) also used other tools including Peabody Picture Vocabulary Test (PPVT), Expressive Vocabulary Test (EVT) and the communication sub-domain of SRS, respectively [69,70]. Neither study found any effect of *n*-3 LCPUFA on communication. Similarly, the Mankad, 2015 study [37] did not find any differences across groups and the Voigt, 2014 study [55] reported worsened outcome (reported by teachers) in response to *n*-3 LCPUFA supplementation compared to placebo that showed improvement (1.4 vs. −4.5, *p* = 0.02). However, the Yui, 2012 study [57] found greater improvements in SRS communication sub-scale scores in *n*-3 LCPUFA group than the placebo group (−23.6 vs. −20.6, *p* = 0.03).

**Repetitive and restricted interests and behaviours:** The fixed mean difference for RRB (assessed using ABC) favoured *n*-3 LCPUFA with small effect (−1.08 [−2.17, −0.01], *Z* = 1.94, *p* = 0.05) (Figure 6C) and nil heterogeneity (*I*^2^ = 0%, *p* = 0.68). Removing the Yui, 2011 study [54] removed the significance (*p* = 0.08) perhaps due to low statistical power. Using the Autism Diagnostic Interview-Revised (ADI-R) scale, Yui et al. (2011) also reported a significant improvement in one of four RRB sub-domains, stereotyped and repetitive motor movement (−1.7 vs. −0.7, *p* = 0.04). However, Bent et al. (2014), using the RRB sub-domain of SRS, reported a trend favouring placebo, −2.9 ± 12.0 (placebo) vs. −8.6 ± 11.4 (*n*-3 LCPUFA), *p* = 0.08 [70]. Neither Yui et al. (2012) (using SRS RRB subscale) nor Mankad et al. (using Pervasive Developmental Disorders Behavioural Inventory (PDDBI) resistance to change subscale) showed an effect of *n*-3 LCPUFA intervention on RRB (*p* > 0.05) [37,57]. The mean scores improved in both treatment groups in these studies.

#### 3.2.2. Effect of LCPUFA on Co-Existing Conditions

**Figure 7:**
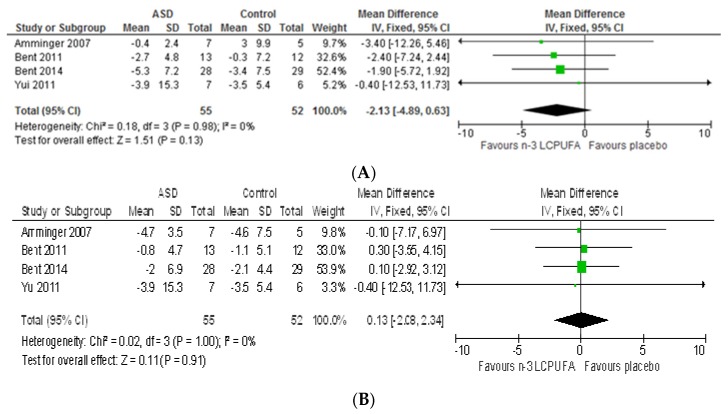
Forest plot of mean (95% confidence interval (CI)) fixed difference in change in hyperactivity (ABC) (**A**) and irritability (ABC) (**B**) in populations with Autism Spectrum Disorder (ASD) receiving *n*-3 long chain polyunsaturated fatty acid supplementation (*n*-3 LCPUFA) and placebo. Direction of effect (negative, more improvement in *n*-3 LCPUFA group; positive, more improvement in placebo group; zero, no difference between groups).

**Hyperactivity:** Hyperactivity scores (assessed using ABC) did not differ between treatment groups (−2.13 [−4.89, 0.62], *p* = 0.13) (Figure 7A) and heterogeneity was nil (*I*^2^ = 0%, *p* = 0.98). Sensitivity analysis by removing studies including older participants [36,54] had no effect on the overall result. Similarly, using BASC hyperactivity sub-domain, the Bent, 2011 study [69] did not find any difference between groups (*p* = 0.83). Using the BASC externalizing behaviour scale, Mankad et al. (2015) reported a significantly worsened outcome in response to *n*-3 LCPUFA supplementation compared to placebo (3.2 vs. −3.0, *p* = 0.02) [37]. It should be noted that BASC externalizing behaviour is a composite measure of hyperactivity, aggression and conduct problem. The authors suggested that greater pre-existing gastrointestinal distress at baseline (8/19 vs. 1/19, in the *n*-3 LCPUFA group vs. placebo group) may have predisposed the *n*-3 LCPUFA group to higher externalizing behaviour.

**Irritability:** Irritability scores (assessed using ABC) did not differ between groups (0.13 [−2.08, 2.34], *p* = 0.91) (Figure 7B) and heterogeneity was nil (*I*^2^ = 0%, *p* = 1.00). Sensitivity analysis by removing studies including older participants [36,54] had no effect on the overall result.

**Sensory issues:** The Mankad, 2015 study [37] was the only study that assessed the effect of *n*-3 LCPUFA supplementation on sensory issues (using sensory/perceptual approach behaviour domain of PDDBI). Sensory symptoms comparably improved in both study groups. It should be noted that this domain has five clusters, all of which tap into a variety of repetitive behaviours.

**Gastrointestinal symptoms:** The Mankad, 2015 study [37] was the only study that assessed the effect of the intervention on gastrointestinal distress and found no differences across treatment groups (*p* > 0.9) (assessed using CGI-I).

Publication bias could not be determined for any outcome measures due to small number of studies included in the meta-analysis (*n* = 4).

#### 3.2.3. Tolerability and Safety of LCPUFA Supplementation

All RCTs included in this review concluded that LCPUFA supplementation was well tolerated and safe. Adverse effects reported were not serious and were comparable across treatment groups.

## 4. Discussion

The findings of each meta-analysis are individually discussed (starting with a discussion of findings of meta-analysis 1 and then meta-analysis 2) followed by a discussion on potential mechanistic pathways that might underlie the relationship between LCPUFA and ASD.

### 4.1. Systematic Review and Meta-Analysis 1

The current study (meta-analysis 1), to our knowledge, is the first meta-analysis of case control studies of blood fatty acid levels in populations with ASD. The findings of this study were that children with ASD had lower levels of DHA, EPA, and higher total *n*-6 LCPUFA to *n*-3 LCPUFA ratio, but not ARA to DHA and ARA to EPA ratios, compared to typically developing children. However, the differences were only evident in studies that included children only and not in studies with wide age ranges that also included adolescents and adults. One should be cautious to make conclusions regarding the modulating effect of age on the relationship since the number of studies including homogenous samples of adolescents and homogenous samples of adults are limited.

Herein we compare the findings of the current study with those of meta-analyses in ADHD because there is an overlap in symptoms between ASD and ADHD. Our results are in agreement with recently published meta-analyses showing lower *n*-3 LCPUFA levels, with larger effect size when DHA and EPA within each study were pooled than when these fatty acids were separately considered [71], and higher ratios of *n*-6 LCPUFA to *n*-3 LCPUFA [42] in patients with ADHD compared with healthy controls.

Another finding of the current study is that while the majority of included studies were of high quality, there was large methodological and clinical heterogeneity between studies, highlighting the importance of discussing the results in light of the study and population characteristics. The type of blood tissue in which the fatty acid composition is analysed has been suggested to affect the findings of case-control studies [27,51]. Bell et al. (2010) and Jory (2016) compared plasma/serum fatty acid composition with those of RBC [27,51]. The authors found significantly lower LA [27], ARA, DHA, and EPA [51] and higher *n*-6 LCPUFA to *n*-3 LCPUFA ratio [51] in RBC of autistic children compared with healthy controls. No polyunsaturated fatty acids in Bell et al.’s study [27] and only DHA in Jory’s study [51] were found to be significantly different across groups when plasma/serum were compared. However, sensitivity analysis in the current study revealed no effect of removing studies that reported RBC fatty acids on heterogeneity.

The method by which blood fatty acid composition is expressed (relative vs. absolute) has also been shown to alter the findings of case-control studies investigating the fatty acid composition across groups and to modify the LCPUFA—disease relationship [46,53]. Yui et al. (2016) compared relative levels of plasma fatty acids with the same fatty acids expressed as absolute [53]. The authors found a significant difference in DHA, EPA, DPA, and arachidic acid, and the ratios of ARA to DHA and ARA to EPA (the reverse ratios were reported) across groups when relative levels were expressed. However, the significance disappeared for DHA and arachidic acid when absolute levels were compared. Although the removal of those studies reporting absolute levels had no impact on the heterogeneity, it resulted in the loss of significance across groups for ARA and in a significantly higher ARA to EPA in populations with ASD than typically developing controls. These findings highlight the importance of taking the method by which blood fatty acid composition is expressed into account when blood fatty acid profile is investigated. A potential explanation for such findings could be the inter-study variation in extraction and separation efficiencies in fatty acid analysis [46].

The high heterogeneity reported in this meta-analysis could also be explained by populations’ characteristics. The role of age and sex on fatty acid status has been well documented [60,72,73]. Using a linear model, Wiest et al. (2009) demonstrated that ARA level was modified by sex; while ARA level did not differ between male autistic children and healthy controls, female autistic children had significantly lower ARA than healthy controls [60]. Thus, an uneven distribution of age (particularly if a wide age range is included) and sex across groups could alter the results in different ways; mask the difference, change the effect direction, or influence the effect size, all of which may contribute to heterogeneity seen in this study. Lack of effect in the Bell, 2004 study (EPA, DHA, and ARA) [66], change of direction in the El-Ansary, 2011a (ARA/EPA and ARA/DHA ratios) and 2011b (ARA), and Sliwinski, 2006 studies (total *n*-3 LCPUFA, DHA, and total *n*-6/*n*-3 LCPUFA ratio) [49,50,65], and the large effect size in the Parletta, 2016 study (EPA, DHA, ARA and their ratios) [52] may be explained by such characteristics.

The use of psychotropic medication can be another modifier. Psychotropic medications such as rispiradone may affect RBC fatty acid composition through their effect on oxidative stress and lipid peroxidation [23,74]. The Parletta, 2016 study [52] together with the Tostes, 2013 study [68] resulted in high levels of heterogeneity in EPA. ASD children in the former study were significantly younger than typically developing children [52], and approximately 88% of children with ASD in the Tostes, 2013 study were on psychotropic medication [68].

Location is another factor that may modify the fatty acid composition–autism relationship. With reference to those studies included in this meta-analysis, the Yui, 2016 [53] and Sliwinski, 2006 [49] were conducted in Japan and Belgium (among European countries) where the habitual dietary intake of fish and fish products are potentially high [75]. Both studies reported the autistic population having significantly higher DHA and EPA levels and lower *n*-6 to *n*-3 LCPUFA ratio than healthy controls, and both affected heterogeneity largely [49,53]. High consumption of *n*-3 LCPUFA rich foods can mask the difference, and in populations who are potentially prone to disturbances in fatty acid metabolism can alter the effect direction [49,53].

Altered fatty acid composition in ASD has been suggested to be, in part, due to low dietary intake of LCPUFA. Children with ASD have very limited food preferences that may result in these children having limited intake of LCPUFA rich foods [67]. Only three studies included in this meta-analysis assessed the dietary intake of fatty acids [14,53,67]. Al-Farsi, 2013 reported a lower intake of ALA, assessed using a semi-quantitative food frequency questionnaire, in children with ASD than healthy controls [0.8 (0.2) vs. 1.2 (0.4) g/day, *p* = 0.001] [67]. However, Ghezzo et al. (2013) [14] and Yui et al. (2016) [53] found no difference in LCPUFA intake across groups, pointing to the facts that these abnormalities could be due to disturbances in fatty acid metabolism rather than intake. For a brief discussion on the potential mechanistic pathways of LCPUFA in ASD refer to Section 4.3 (Potential mechanistic pathways).

### 4.2. Systematic Review and Meta-Analysis 2

Few RCTs have been completed and reported to date on *n*-3 LCPUFA supplementation for ASD; only six trials were included in this review (four included in the meta-analysis and two in the overall interpretation), with a total of 178 participants. In this meta-analysis, a small but significant benefit of *n*-3 LCPUFA supplementation was found for social interaction and RRB but not communication and co-existing behaviours and conditions. No evidence of significant heterogeneity between trials were found.

Four trials included in the present meta-analysis with a total of 107 participants are however insufficient to provide robust evidence. Furthermore, the findings cannot be generalised to all children on the autism spectrum because the included children predominantly comprised males, were of different age groups (less than eight years and up to 28 years), displayed moderate to severe symptoms [54,69] or high hyperactivity level [36,70].

The findings of this review to some extent contradict the results of previous systematic reviews and meta-analysis [38,39]. These reviews did not find any statistically significant improvement in behaviour, but reported a larger positive effect on hyperactivity [38,39] while the present review found a significant improvement in social interaction and no improvement in hyperactivity. Our results, however, are in line with those of previous reviews [38,39] in that no improvement was identified in communication and irritability. With respect to RRB, sensory issues and gastrointestinal symptoms, no comparison can be made because they were not considered by these reviews. It should be noted that the small number of RCTs included in the review by Bent et al. (*n* = 1) [38] and James et al. (*n* = 2) [39] could have compromised the statistical power to detect any difference across groups.

Although case-control and open label studies provided evidence for a role of *n*-3 LCPUFA in ASD, RCTs of supplementation with *n*-3 LCPUFA yielded mixed results. One reason for such inconsistencies between studies may result from inadequately controlling for age, trial duration, habitual dietary intake of *n*-3 LCPUFA and levels of these fatty acids in the circulation over the course of trial, participants’ general health conditions at the baseline and over the course of study, and outcome tools assessing behaviour. Response to *n*-3 LCPUFA supplementation has been shown to be predicted by body weight adjusted dose, baseline omega-3 index (RBC DHA + EPA), sex, age, and physical activity level; with populations receiving larger doses, having lower starting omega-3 index, older population, females and those with higher physical activity level experiencing a greater increase in the omega-3 index in response to supplementation [76].

Dietary intake of *n*-3 LCPUFA rich foods in children with autism is low [67]. However, omega-3 fatty acid supplements are among the most commonly used complementary and alternative medication in ASD [40]. It is plausible to suggest that even though the mentioned trials excluded participants that used *n*-3 LCPUFA supplements on their own initiatives at baseline, participants may have had high habitual dietary intake of these fatty acids due to their popularity, therefore responding differently to supplements which leads to diminishing the differences across treatment groups. An example of such implication could be the Voigt, 2014 study [55]; all children in this study had baseline LCPUFA levels above paediatric reference ranges for nutritional deficiencies and metabolic disorders (established at the Mayo Clinic [77]), and despite an increase of 431% in plasma DHA level, no improvement in behaviour was reported. Bent et al. (2011), on the other hand, showed that higher baseline level of some *n*-6 and *n*-3 polyunsaturated fatty acids including eicosadienoic acid (*r* = −0.79, *p* = 0.02), docosadienoate acid (*r* = −0.65, *p* = 0.03), and ALA (*r* = −0.64, *p* = 0.03) were associated with reduction in hyperactivity [69]. In an open label trial of *n*-3 LCPUFA in 41 autistic children aged 7–18 years, Ooi et al. (2015) showed an inverse correlation between autism mannerism severity and change in RBC fatty acids after 12 weeks of intervention and the severity was associated with baseline EPA level [32]. Unfortunately, the number of trials included in this review was inadequate to provide data on the effect of baseline *n*-3 LCPUFA intake or status on behavioural changes in response to supplementation.

The sex differences in ASD might be partly explained by sex differences in fatty acid metabolism; males may be more vulnerable than females to deficiencies in LCPUFA because of hormonal reasons [78], and thus may respond poorly to supplements [76]. With the exception of one study [36], no trials included in this review stratified the randomisation by sex. Unfortunately, no analysis could be performed for subgroups stratified by sex in this review because far more males than females were included and no studies reported behavioural changes in response to supplementation for males and females separately. With regard to the potential effect of age, three out of six studies included a wide age range (children and young people up to 28 years) which may have resulted in greater response variability. Due to the small number of studies included in this review, we could not perform subgroup analysis for different age bands to examine the effect of age on behavioural change in response to omega-3 supplementation.

The trial duration varied widely in studies included in this review (6–26 weeks). Evidence suggests that PUFA erythrocyte membrane reaches a steady state after 6 months [79] and at least 4 months is needed to demonstrate an effect on cognitive performance [41]. It has also been suggested that longer study periods of one year might be needed to demonstrate behavioural changes in response to *n*-3 LCPUFA supplementation [80]. Furthermore, the majority of outcome assessment tools are retrospectively completed (and duration over which behaviour is assessed varies depending on the assessment tool used); for example, parents are required to consider the behaviour over the past four weeks when completing ABC questionnaire while the timeframe for SRS is the past six months. It is of importance to avoid assessing and considering behaviour when the LCPUFA erythrocyte membrane has not yet reached its steady state. Therefore, wide variation and insufficient trial duration could explain the inconsistencies. Due to the small number of studies included in this review, we could not perform subgroup analysis for different intervention lengths to examine the effect of duration on behavioural change in response to omega-3 supplementation.

Gastrointestinal symptoms are highly prevalent in populations with ASD [81,82,83]. Mazurek et al. (2013) reported that of 2973 children with ASD, 24.7% had at least one chronic gastrointestinal symptom [83]. Compared to typically developing children, developmentally delayed and ASD children were more likely to have at least one frequent gastrointestinal symptom [83]. ASD children with frequent abdominal pain, gaseousness, diarrhea, constipation or pain on stooling had worse scores in four (irritability, social withdrawal, stereotypy, and hyperactivity) out of five ABC subscales than ASD children with no frequent gastrointestinal symptoms [84]. The pain and discomfort caused by gastrointestinal distress can worsen the behaviour in people with ASD, more particularly in non-verbal individuals who cannot express their feelings. Mankad et al. (2015) reported significantly worsened externalizing behaviours in response to *n*-3 LCPUFA supplementation which could be attributed to higher gastrointestinal distress reported in the active treatment group compared with the placebo (8/19 vs. 1/19, respectively) [37]. Thus, it is important to consider the potential modulating effect of gastrointestinal symptoms over the course of study on behavioural changes in response to supplementation.

Additionally, a variety of assessment tools (ranging from one to six tools) were used in each study which complicated effective comparisons across studies resulting in the exclusion of three studies from the meta-analysis, and potentially compromised the validity of a study by increasing the likelihood of type 1 error [44]. With the exception of ABC, a widely-used tool to assess problem behaviours in pharmacological trials in ASD, the majority of assessment tools have been designed for diagnostic purposes and there is a lack of evidence regarding the sensitivity of these tools to slight changes in behaviour in response to intervention in ASD populations. It is also worth noting that the inappropriate speech subscale of ABC is not a comprehensive measure of communication compared to other tools like SRS (inappropriate speech subscale of ABC comprises of four questions while the SRS communication subscale consists of 22 questions). Thus, findings regarding communication (measured by ABC) should be interpreted with caution.

It is also worth noting that the small or lack of effect reported here could be due to large placebo response in many trials included in this review. The large placebo response may have limited our capacity to identify any differences in some behaviour across groups. It is documented that different factors [e.g., raters of outcome assessment tools (clinicians vs. parents), increased response to active intervention, location, pharmacological and adjunctive intervention, participants’ age (younger vs. older), study duration (shorter vs. longer), and severity of condition (lower baseline severity vs. higher severity)] are associated with the increased placebo response in ASD and other neurodevelopment and psychiatric disorders [85,86,87]. However, the reason for observed improvement in placebo groups included in this review is unclear and could not be determined due to small number of studies included.

### 4.3. Potential Mechanistic Pathways

There are, though not very well understood, several potential biological pathways for a role of LCPUFA in ASD [88,89]. Approximately 60% of the brain’s dry weight is fat, with DHA comprising 60% and 40% of the PUFA in the retina and brain, respectively [90], suggesting that it is structurally important. Evidence suggests that some individuals with ASD have abnormalities in the gray and white matter of brain regions that are involved in social interaction, RRB, and sensory processing [13,91,92,93]. Ingestion of DHA (through diet or supplementation) has been shown to be positively associated with gray matter volume and its functional integrity and with white matter microstructural integrity in healthy individuals [16,17,94].

At the cellular level, PUFA interact with and influence the functioning of integral membrane proteins, including enzymes, receptors, and ion channels [95,96]. Evidence suggests that the activity of Na^+^/K^+^-ATPase (an enzyme that controls ion transport produced by neurotransmission) and adenylate cyclase (an enzyme that catalyses the conversion of ATP to cyclic AMP and has been shown to modulate social behaviours [97]) is disturbed in individuals with ASD [14,98]. Ghezzo et al. (2013) reported children with ASD having a significant reduction in Na^+^/K^+^-ATPase activity, alterations in erythrocyte fatty acid membrane (a decrease in *n*-3 LCPUFA and consequently an increase in *n*-6 LCPUFA to *n*-3 LCPUFA ratio, and an increase in monounsaturated fatty acids), and a reduction in erythrocyte membrane fluidity [14]. These alterations correlated with clinical features of ASD, particularly hyperactivity scores [14].

Further evidence for a relationship between *n*-3 LCPUFA and ASD comes from studies investigating its role in neurogenesis and several neurotransmitter systems. LCPFA, particularly *n*-3 series DHA, has been shown to favourably affect neurite survival, outgrowth and myelination in animal cultured cortical [99,100,101], sensory [102], and hippocampal neurons [18,103,104]. The development of axons and dentrites as well as myelination in multiple brain areas (involved in social behaviours, emotions, and RRB) has been reported to be impaired in individuals with ASD [105,106]. Similarly, an abnormal level of brain derived neurotrophic factor (BDNF, a protein that promotes the survival of neurons) in the circulation has been reported in children with ASD, which was associated with the severity of condition [88,107,108]. Docosahexaenoic acid administration normalised BDNF in the hippocampus, increased the growth of uninjured corticospinal and serotonergic fibres, and enhanced synaptic plasticity in an animal model of spinal cord injury [101,109].

Autistic children have been shown to exhibit significantly higher levels of several dopamine derivatives (in urine), dopamine transporter binding proteins, and serotonin in brain, and lower levels of serotonin transporter binding protein (in brain), glutamine signal (in basal ganglia), and oxytocin than healthy controls [110,111,112,113,114,115,116]. Furthermore, within the ASD populations, basal ganglia glutamine signal and plasma oxytocin negatively correlated with impaired behaviour [114,116]. In response to an *n*-3 PUFA limited diet in rats, dopamine levels reduced and basal synaptic release of serotonin increased while the turn over metabolites of dopamine increased and those of serotonin decreased [19,117,118,119]. A DHA depleted diet also altered the glutamergic system in offspring female rats [120]. This alteration was associated with anxiety-like behaviours, memory deficit and exploratory behaviours during adulthood [120]. On the other hand, DHA treatment significantly increased synaptic plasticity in hippocampal neurons and enhanced glutamatergic activity [121].

Another plausible mechanism supporting the association between LCPUFA and ASD is the anti- and pro-inflammatory properties of LCPUFA metabolic products. Eicosanoids (a collective name for prostaglandins, thromboxanes, leukotrienes and a variety of hydroxyl and hydroproxy fatty acid) are the enzymatic metabolic products of PUFA, and have important roles in inflammation [20]. While EPA or DHA derived eicosanoids have anti-inflammatory properties, those derived from ARA have pro-inflammatory properties [20]. Elevated levels of several peripheral pro-inflammatory cytokines and nuclear factor Kappa B (NF-κB, a transcription factor involved in inflammatory signaling pathways) has been reported in children with ASD [122,123,124]. Brigandi et al. [24] reported children with ASD having significantly higher plasma levels of PGE2 than healthy controls, a finding confirmed by El-Ansary and Al-Ayadhi (2012) [125] who also reported higher levels of leukotriene and 8-isoprostane together with PGE2 in children with ASD. In addition, lower levels of antioxidant proteins and increased levels of oxidative stress markers was associated with more severe ASD symptoms, including sensory issues [14,124,126]. Supplementation with *n*-3 PUFA, on the other hand, decreased the gene expression of NF-κB, IL-12 and IL-13 [127], macrophage inflammatory protein-2 (MIP2), IL-6 [128] and tumor necrosis factor-α (TNF-α) [128,129,130].

Decreased antioxidant capacity and increased lipid peroxidation may result in RBC LCPUFA instability and decrease these fatty acids in autism [23,74]. Instability in RBC LCPUFA composition has been shown by a great loss in PUFA levels when the blood samples of autistic children were stored at −20 °C, a finding not observed in the blood sample of healthy controls [62]. The reason for such instability could be related to cellular phospholipase activity. Tostes et al. (2013) [68] and Bell et al. (2004) [66] reported children with ASD having significantly higher phospholipase A2 (PLA2) activity than typically developing controls that was reduced by EPA supplementation [66]. PLA2 is responsible for releasing fatty acids, more particularly ARA, from phospholipids [66].

Finally, the role of LCPUFA in ASD could be explained by defects in enzymes involved in the conversion of LCPUFA from their precursors or deficits in the process of incorporation of LCPUFA into the cell membrane [24,25,26]. Gene variants in fatty acid desaturase (FADS)—one of the strongest genome wide associated signals—have been shown to enhance the conversion of ARA from its precursor and to be sex- and ethnicity-specific [131,132]. The effect of FADS genotype has been shown to be more pronounced in African Americans than Europeans (approximately two fold higher) [131]. The higher frequency of this genetic variant, but not the allelic effect of G allele, explained such a difference [131]. Also, while in Caucasians, one FADS2 single nucleotide polymorphism (SNP) and multiple FADS2 SNP (two SNP in males and nine SNP in females) were associated with *n*-6 aggregate desaturase indices, it was associated with five FADS2 SNP in East Asian Females [132]. In addition, carriers of APOE4 allele seems to have altered long chain omega-3 metabolism [133]. Compared to the non APOE4-carriers, the carriers have higher β-oxidisation rates of *n*-3 LCPUFA [133]. Furthermore, Shimamoto et al. (2014) showed altered mRNA gene expression levels of fatty acid binding protein 7 (FABP7) in post-mortem ASD brains, and increased hyperactivity and anxiety-related phenotype (two common features in ASD) in FABP7 knockout mice [134]. Although the modifying role of genetic variants in enzymes involved in fatty acid metabolism in the LCPUFA-disease relationship has been well documented, in the context of ASD, it warrants further investigation.

## 5. Conclusions

The current meta-analysis of case-controls studies, to our knowledge, is the first to investigate fatty acid composition in populations with ASD. Future observational studies of *n*-3 LCPUFA in children with ASD are encouraged while including a uniform biomarker (e.g., omega-3 index or percentage of *n*-3 LCPUFA in RBC) and reporting method (e.g., relative or absolute), collecting dietary intake of both *n*-3 and *n*-6 LCPUFA, and matching cases and controls on potential modulating attributes (e.g., age, sex, severity, genotype, and medication use). It is also critical to know whether inadequate LCPUFA status in ASD is attributed to inefficient or disrupted metabolism or other factors like LCPUFA consumption.

Based on the current evidence, *n*-3 LCPUFA supplementation cannot be recommended as an alternative to support behavioural therapies for ASD children. However, it seems prudent that *n*-3 LCPUFA could be used to complement other therapies in ASD populations given its long-term tolerability and acceptability (up to six months), potentially inefficient or disrupted LCPUFA metabolic pathways in this population, and its critical role in brain function and development, and various body processes some of which are involved in the pathobiology of ASD. It should be noted that this recommendation is made cautiously because the results of this study are based on a very small sample of studies (with methodological differences and limitations) and short duration of the interventions. Therefore, the generated statistics should not be over-interpreted but seen as indicative of the need to study the issue further (while controlling for potential modifying and confounding variables) to pursue the trends observed in this study. The effect of *n*-3 LCPUFA on behaviour may be modulated by background diet and baseline LCPUFA status, sex, age, trial duration, and gastrointestinal stress at baseline and over the study period, all of which are recommended to be considered in future research. Furthermore, an investigation of the potential modulating effect of genotype (e.g., APOE) on behavioural changes in response to LCPUFA supplementation is warranted. It is also recommended that future studies include a uniform assessment tool that is sensitive to minor behavioural changes in response to complementary/nutritional therapies. Finally, the potential placebo effect and the reasons for such effects are encouraged to be investigated and accounted for in the design stage of RCTs.

## Figures and Tables

**Table 1:** Characteristics of case-control studies included in meta-analysis 1.

Reference and Setting	Cases Characteristics				Controls Characteristics				Matching	Outcome				Quality Score †
	*N*	Condition, Classification System, Tools ᵟ	Age (Years) *	Sex (M, F)	*N*	Health Condition	Age (Years) *	Sex (M, F)		Blood Tissue Type	Fasting State (Length)	Values Reported as	FA and Ratios Compared and the Direction of Difference	
Al-Farsi (2013) [67] Oman	40	Autism, based on DSM-IV, NR	4.1 (0.9)	NR	40	Healthy and TD	4.1 (0.8)	NR	Age Sex	Serum	NR	µg/mL	DHA ↓	6
Bell (2004) [66] UK	29	11 classical autism and 18 regressive autism, NR, NR	NR	NR	55	Healthy and TD	NR	NR	None	RBC	NR	% of total FA	Total *n*3 ↓ DHA ↓ EPA ↓ Total ↔ ARA ↔ ARA/EPA ↑	4
Bell (2010) [27] UK	45	Autism, based on DSM-IV and ICD-10, ADI-R	7.5 (3.5)	39 M, 5 F	52	Healthy and TD	7.5 (3.6)	49 M, 3 F	Age Sex (45 pairs matched)	RBC Plasma	NR	% of total FA	Total *n*3 1 ↔ DHA ↔ EPA ↔ Total ↔ ARA ↔ Total *n*6/*n*3 2↑ ARA/EPA ↑	8
Brigandi (2015) [24] US	121	Autism (but not Asperger or PDD-NOS), based on DSM-IV, CARS	3–17 **	NR	110	Non autistic and developmentally delayed	3–17 **	NR	NR	RBC	No	% of total FA	Total *n*3 ↓ DHA ↓ EPA ↔ Total *n*6 ↓ ARA ↓ Total *n*6/*n*3 2↑	6
Bu (2006) [28] US	40	Autism and regressive autism, based on DSM-IV and ICD-10, ADI-R and ADOS	3.6 ***	37 M, 3 F	20	TD	3.5 ***	16 M, 4 F	Age Sex Geographical residential area	RBC	Yes (2 h)	% of total FA	Total *n*3 ↔ DHA ↔ EPA ↔ Total ↔ ARA ↔ ARA/EPA ↔	8
El-Ansari (2011a) [50] Saudi	25	Autism, NR, ADI-R, ADOS, 3di	4–12 **	NR	16	Healthy and TD	4–11 **	NR	Age	Plasma	Yes (10 h)	mmol/L	ARA/DHA ↓	7
El-Ansari (2011b) [65] Saudi Arabia	22	Autism, NR, ADI-R, ADOS, 3di	4–12 **	NR	26	Healthy and TD	4–11 **	NR	Age	Plasma	Yes (10 h)	mmol/L	DHA ↓ EPA ↔ ARA ↓	7
Ghezz (2013) [14] Italy	21	Autism, DSM-IV, ADOS and CARS	6.8 (2.2)	17 M, 4 F	20	Healthy and TD	7.6 (1.9)	14 M, 6 F	Age Sex	Serum	NR	% of total FA	DHA ↓ EPA ↓ ARA ↔ Total *n*6/*n*3 ↑	11
Jory (2016) [51] Canada	11	Autism, DSM (version NR), NR	3.9 (1.7)	8M, 3F	15	Healthy and TD	3.9 (1.1)	6 M, 9 F	Age	RBC Serum	Yes (NR)	% of total FA	DHA 1 ↓ EPA ↓ ARA ↓ Total *n*6/*n*3 ↑ ARA/DHA ↔ ARA/EPA ↔	7
Meguid (2008) [30] Egypt	30	Autism, DSM-IV, clinical evaluations and CARS	3–11 **	18 M, 12 F	30	Healthy and TD	NR	NR	Age Sex	Whole blood	NR	µg/mL	DHA ↓ ARA ↓ ARA/DHA ↓	7
Mostafa (2015) [26] Egypt	80	Autism, DSM-IV, clinical evaluation and CARS	7.4 (3.3)	66 M, 14 F	80	Healthy and TD	7.3 (3.1)	66 M, 14 F	Age Sex	Plasma	NR	mmol/L	DHA ↓ ARA ↓ ARA/DHA ↑	8
Parletta (2016) [52] Australia	85	Autism, clinical evaluation and CARS	5.3 (2.1) ^	68 M, 17 F	79	Healthy and TD	8.3 (2.5) ^	61 M, 18 F	Sex	RBC	NR	% of total FA	DHA ↓ EPA ↓ ARA ↓ Total *n*6/*n*3 ↑ ARA/EPA ↑	9
Sliwinski (2006) [49] Belgium	18	Autism with IQ > 55 and post pubertal, DSM-IV, ADI-R	12–20 **	Only male	22	TD post pubertal	12–22	Only male	None	Plasma	Yes (overnight)	% of total FA	Total *n*3 ↑ DHA ↑ EPA ↔ Total *n*6 ↔ ARA ↔ Total *n*6/*n*3 2↓	8
Tostes (2013) [68] Brazil	24	Autism, DSM-IV, clinical evaluation	7.4 (2.9)	18M, 6F	24	Healthy and TD	7.2 (1.8)	18 M, 6 F	Age sex	Plasma	NR	% of total FA	DHA ↓ EPA ↓ ARA ↑ ARA/DHA 3 ↑ ARA/EPA 3 ↑	9
Yui (2016) [53] Japan	28	Autism with IQ/70, DSM-IV, ADI-R	13.5 (4.7)	20M, 8F	21	Healthy and TD	13.9 (5.7)	15 M, 6 F	Age Sex IQ Eating habit Home environment	Plasma	NR	% of total FA and µg/mL	DHA 4 ↑ EPA ↑ ARA ↓ ARA/DHA ↓ ARA/EPA ↓	8

ᵟ Psychological assessment tools used to confirm ASD diagnosis. * Reported as mean (SD) unless otherwise stated. † Health Canada Quality Appraisal Tool for Observational Studies; A quality score of ≥7 was considered higher quality [47]. ** Inclusion criteria. *** Median. ^ Significantly different. ↓ Cases had lower levels than controls (*p* < 0.05). ↑ Cases had higher levels than controls (*p* < 0.05). ↔ No difference across groups (*p* > 0.05). ^1^ RBC values are reported. ^2^ A borderline significance. ^3^ The significance was not reported. ^4^ % of total fatty acids is reported. ADI-R, Autism Diagnostic Interview-Revised; ADOS, Autism Diagnostic Observation Schedule; ARA, arachidonic acid; CARS, Childhood Autism Rating Scale; DHA, docosahexaenoic acid; DSM-IV, Diagnostic and Statistical Manual of Mental Disorder-Fourth Edition; EPA, eicosapentaenoic acid; F, female; FA, fatty acids; M, male; N, number of participants; NR, not reported; RBC, red blood cell; TD, typically developing; 3di, the Developmental, Dimensional, and Diagnostic Interview.

**Table 2:** Study characteristics of randomised controlled trials (RCTs) included in systematic literature review.

**RCTs included in Meta-Analysis 2 (*n* = 4)**									
**Reference and Setting**	**Age (Years)**	**Sex Distribution (M, F)**	**Sample size**	**Intervention**		**Duration**	**Outcome Measure**	**Outcome**	**Quality Score †**
				**Active**	**Placebo**				
Amminger (2007) [36] Austria Pilot	5–17	All male	Intervention (*n* = 7) Placebo (*n* = 6, 1 lost)	0.84 g/day EPA 0.7 g/day DHA	7 g/day coconut oil	6 weeks	ABC	No significant differences between groups at 6 weeks, but a greater change in hyperactivity and stereotypy subscale with a large effect size in the omega-3 group than placebo (7 and 2.4 units, effect size of 0.71 and 0.72, respectively). Well tolerated and safe.	10
Ben (2011) [69] US Pilot	3–8	24 M, 3 F	Intervention (*n* = 14, 1 lost and 4discontinued) Placebo (*n* = 13, 1 lost and 2 discontinued)	0.7 g/day EPA 0.46 g/day DHA	Orange flavoured pudding containing safflower oil	12 weeks	ABC PPVT EVT BASC SRS CGI-S	Significant increase in the percentage of serum omega-3 fatty acids. No significant differences in all measure across groups. Non-significant greater improvement in hyperactivity subscale in omega-3 group than placebo (2.7 vs. 0.3 units, effect size of 0.38). Decreases in some fatty acids correlated with decreased in hyperactivity. Well tolerated and safe.	13
Bent (2014) [70] US Internet-based	5–8	50 M, 7 F	Intervention (*n* = 29, 8 discontinued and 2 improper enrolment) Placebo (*n* = 28, 4 discontinued and 1 improper enrolment)	0.7 g/day EPA 0.46 g/day DHA	Orange flavoured pudding containing safflower oil	6 weeks	ABC-(parent and teacher) SRS CGI-I	No significant differences in changes in SRS and CGI-I between groups. Non-significant greater improvement in hyperactivity subscale in omega-3 group than placebo (−5.3 vs. −3.4, effect size of 0.26). Significantly greater improvements in stereotypy and lethargy subscales (*p* = 0.05 and 0.01, respectively). Well tolerated and safe.	13
Yui (2011 and 2012) * [54,57] Japan Pilot	6–28	12 M, 1 F	Intervention (*n* = 7) Placebo (*n* = 6)	0.24 g/day DHA 0.24 g/day AA	Olive oil	16 weeks	ABC ADI-R SRS	Significant increase in plasma AA. No differences in plasma DHA and EPA. Significant improvement in social withdrawal subscale of ABC (*p* = 0.04) and stereotyped and repetitive behaviours of ADI-R (*p* = 0.04). Significant improvement in communication subscale of SRS reported in both treatment and placebo groups, though the effect size was more favourable for the treatment group than placebo group (0.87 vs. 0.44, respectively). Safe.	10
**RCTs Not included in the Meta-Analysis 2 but included in the Overall Interpretation (*n* = 2)**									
**Reference and Setting**	**Age (Years)**	**Sex Distribution (M, F)**	**Sample Size**	**Intervention**		**Duration**	**Outcome Measure**	**Outcome**	**Quality Score †**
				**Active**	**Placebo**				
Mankad (2015) [37] Canada	2–5	27 M, 10 F	Intervention (*n* = 19, 4 drop outs) Placebo (*n* = 19, 2 drop outs) Stratified by severity	1.5 g/day EPA + DHA	Refined olive oil in medium chain triglyceride	6 months	PDDBI BASC-2 CGI-I VABS-II PLS-4	No significant differences between groups in all measures at 6 months, but mild improvement in BASC-2 externalising subscale in placebo but worsening in omega-3 group (−3 vs. 3, respectively, *p* = 0.02) Relatively well tolerated and safe.	13
Voigt (2014) [55] US	3–10	40 M, 8 F	Intervention (*n* = 24, 5 discontinued) Placebo (*n* = 24, 9 discontinued) Stratified by age	0.2 g/day DHA	0.25 g/day corn oil + 0.25 g/day soybean oil	6 months	CGI-I CDI ABC BASC	431% increase in plasma phospholipid DHA No significant differences in the percentage with a positive response (CGI-I) across groups and in all other measures across groups. Well tolerated and safe.	13

† Health Canada Quality Appraisal Tool for Experimental Studies; A quality score of ≥8 was considered higher quality [47]. * Different outcomes from the same group of participants were reported in two different papers. AA, arachidonic acid; ABC, Aberrant Behaviour Checklist; ADI-R, Autism Diagnostic Interview-Revised; BASC, Behaviour Assessment System for Children; CDI, Child Development Inventory; CGI-I, Clinical Global Impression-Improvement; CGI-S, Clinical Global Impression-Severity; DHA, docosahexanoic acid; EPA, eicosapentanoic acid; EVT, Expressive Vocabulary Test; F, Female; M, Male; n, Number; PDDBI, Pervasive Developmental Disorders Behavioural Inventory; PLS-4, Preschool Language Scale; PPVT, Peabody Picture Vocabulary Test; SRS, Social Responsiveness Scale; US, United States; VABS-II, Vineland Adaptive Behaviour Scale.

## Supplementary Materials

The following are available online at http://www.mdpi.com/2072-6643/9/2/155/s1, Table S1: Study characteristics of open label trials and case-studies excluded from systematic literature review; Table S2: Quality appraisal of included case-control studies; Table S3: Quality appraisal of included RCTs; Figure S1: Risk of bias table showing judgments on each risk factor for each primary study included in both meta-analysis and overall interpretation.
