# Supplementary material for: Relationship between Long Chain n-3 Polyunsaturated Fatty Acids and Autism Spectrum Disorder: Systematic Review and Meta-Analysis of Case-Control and Randomised Controlled Trials
Source: Nutrients. 2017 Feb 19;9(2):155. doi: 10.3390/nu9020155 (PMC5331586; doi:10.3390/nu9020155)
Supplement: Supplementary file 1 [file nutrients-09-00155-s001.pdf]

Supplementary Materials: Relationship between Long Chain *n*-3 Polyunsaturated Fatty Acids and Autism Spectrum Disorder: Systematic Review and Meta-Analysis of Case-Control and Randomised Controlled Trials

Hajar Mazahery, Welma Stonehouse, Maryam Delshad, Marlena C. Kruger, Cathryn A. Conlon, Kathryn L. Beck and Pamela R. von Hurst

Table S1. Study characteristics of open label trials and case-studies excluded from systematic literature review.

Open label trials (*n* = 6)							
**Reference and setting**	**Age (years)**	**Sex distribution (M, F)**	**Sample size**	**Intervention**	**Duration**	**Outcome measure**	**Outcome**
Johnson (2010) [29] US	1–5	NR	*n* = 10 (Intervention group), *n* = 13 (Healthy diet control group)	0.4 g DHA	3 months	CBCL DBOM Mullen's Scales of Early Learning	Significant improvement in externalising subscale of CBCL was reported in children in the DHA group. Significant improvement in affective subscale of CBCL was reported in children on the healthy diet. No other differences were reported. Well tolerated.
Meguid (2008) [30] Egypt	3–11	18M, 12F	*n* = 30	0.028 g EPA<br>0.012 g DHA<br>0.024 g LA<br>0.01 g AA	3 months	CARS	Significant improvement in whole blood omega-3 and -6 levels Significant improvement in autistic behaviour (concentration, eye contact, language development, motor skills) in 20 children DHA levels correlated negatively with CARS in 10 non-respondent children.
iri (2009) [31] Israel	4–7	NR	*n* = 10, 1 drop out	0.38 g EPA<br>0.18 g DHA	12 weeks	CGI ATEC CPRS	8/9 children showed an average improvement of 33% as measured by ATEC and one child did not respond at all. No adverse effect.
Ooi (2015) [32] Singapore	7-18	36M, 5F	*n* = 41	0.19 g EPA<br>0.84 g DHA	12 weeks	SRS CBCL	Significant increase in percentage of EPA and DHA and significant decrease in AA/EPA ratio. Significant improvement in total (-21 units) and all subscales of SRS (2-7

				0.144 g LA<br>0.066 g AA			units, P<0.01 for all and medium to large effect sizes) and in social problem subscale of CBCL (P=0.02 and medium effect size). Change in RBC fatty acids correlated negatively with autism mannerism severity and higher baseline EPA was associated with a better response. Well tolerated.
Patrick (2005) [33] NR	3-10	NR	*n* = 22, 4 drop outs	0.25 g omega-3<br>0.04 g omega-6	90 days	ABBLS	Significant increase in language development and learning skills.
Politi (2008) [34] Italy	18-40	15M, 4F	*n* = 19	0.93 g EPA + DHA	6 weeks	RBC	No significant improvement in problem behaviours and their severity.
**Case study (*n* = 1)**							
Johnson (2003) [35] US	11	1M	*n* = 1 case-study	3 g omega-3 (0.54 EPA) *	4 weeks	Clinical observation	Significant improvement in anxiety, agitation and quality of life

* The amount of other omega-3 fatty acids was not reported AA, arachidonic acid; ABBLS, Assessment of Basic Language and Learning Skills; ATEC, Autism Treatment Evaluation Checklist; CARS, Childhood Autism Rating Scale; CBCL, Childhood Behaviour Checklist; CPRS, Children's Psychiatric Rating Scale; DBOM, direct behaviour observation measure; DHA, docosahexanoicacid; EPA, eicosapentanoicacid; F, Female; LA, Linoleic acid; M, Male; *n*, Number; NR, not reported; RBC, Rossago Behavioural Checklist; US, the United States.

Table S2. Quality appraisal of included case-control studies †.

Reference	Inclusion & exclusion	Attrition		Exposure		Health outcome		Blinding	Comparability of study groups	Statistical significance of trend	Confounders		Total	Confounders not controlled for
		Reported	Reasons	Methodology	Repeated measurement*	Methodology	Verification				Participants demographics	Other risk factors		
Al-Farsi (2013) [67]	1	0	0	1	0	0	0	1	1	1	1	0	6	Cases had lower frequency intake of high DHA foods, lower intake of ALA, lower energy intake, and shorter duration of breastfeeding (all were not considered in statistical analysis), medication use (NR)
Bell (2004) [66]	0	0	0	1	0	0	0	1	1	1	0	0	4	Age, sex, intake of LCPUFA, medication and supplement use (all NR)
Bell (2010) [27]	1	1	1	1	0	1	0	1	1	1	1	0	9	Dietary intake of LCPUFA (NR)
Brigandi (2015) [24]	1	0	0	1	0	1	0	1	0	1	0	0	5	Age, sex, dietary intake of LCPUFA, supplement and medication use
Bu (2006) [28]	1	0	0	1	0	1	1	1	1	1	1	0	8	Dietary intake of LCPUFA, medication use
El-Ansari (2011a) [50]	1	0	0	1	0	1	1	1	1	1	0	0	7	Sex, dietary intake of LCPUFA, medication and supplement use
El-Ansari (2011b) [65]	1	0	0	1	0	1	1	1	1	1	0	0	7	Sex, dietary intake of LCPUFA, medication and supplement use
Ghezzo (2013) [14]	1	1	1	1	0	0	1	1	1	1	1	0	9	Dietary intake of LCPUFA, medication use (NR)

Jory (2016) [51]	1	1	1	1	0	1	0	1	0	1	0	0	7	Sex, dietary intake of LCPUFA
Meguid (2008) [30]	1	0	0	1	0	1	1	1	1	1	1	0	7	Dietary intake of LCPUFA, medication and supplement use
Mostafa (2015) [26]	1	0	0	1	0	1	1	1	1	1	1	0	8	Dietary intake of LCPUFA
Parletta (2016) [52]	1	1	1	1	0	1	1	1	1	1	0	0	9	Age, dietary intake of LCPUFA
Sliwinski (2006) [49]	1	0	0	1	0	1	1	1	1	1	1	0	8	Supplement use, dietary intake of LCPUFA
Tostes (2013) [68]	1	1	0	1	0	1	0	1	1	1	1	0	8	Medication use, dietary intake of LCPUFA
Yui (2016) [53]	1	0	0	1	0	1	1	1	1	1	1	1	9	-

† Health Canada Quality Appraisal Tool for Observational Studies; A quality score of ≥ 7 was considered higher quality [47]. * Measuring the exposure in duplicate or more is of no relevance for case-control studies and therefore all studies received a score of "0" for this criterion. Accordingly, a total score of 11 has been employed for this review instead of using a total score of 12.LCPUFA, long chain polyunsaturated fatty acids; NR, not reported.

Table S3. Quality appraisal of included RCTs †.

Reference	Inclusion and exclusion	Group allocation: Described as randomised	Group allocation: Randomisation	Group allocation: Randomisation appropriateness	Group allocation: Randomisation	Blinding: Participants	Blinding: Researchers	Attrition: Reported numerically	Attrition: Reasons	Intervention: Type described	Intervention: Amount described	Methodology to measure the health effect	Statistical analysis: Between group statistical analysis	Statistical analysis: Intention to treat	Potential confounders	Total	Confounders not controlled for
Amminger (2007) [36]	1	1	0	0	0	1	1	1	1	1	1	1	1	0	0	10	Age, dietary intake or LCPUFA status, higher hyperactivity in omega-3 group, compliance (NR)
Bent (2011) [69]	1	1	1	1	1	1	1	1	1	1	0*	1	1	1	0	13	Medical regimen
Bent (2014) [70]	1	1	1	1	1	1	1	1	1	1	0*	1	1	1	0	13	Sex (the distribution across groups NR), medical regimen, dietary intake or LCPUFA status
Mankad (2015) [37]	1	1	1	1	1	1	1	1	1	1	1	1	1	1	0	14	Gastrointestinal distress
Voigt (2014) [55]	1	1	1	1	1	1	1	1	1	1	1	1	1	0	0	13	Sex (the distribution across groups NR), medical regimen, compliance raw data (NR)
Yui (2011 & 2012) * [54,57]	1	1	1	0	1	1	0	1	1	1	1	1	1	0	0	11	Age, compliance (NR)

† Health Canada Quality Appraisal Tool for Experimental Studies; A quality score of >7was considered higher quality [47]. * Different outcomes were reported in two different papers. *The intervention material was delivered in a pudding form – no information regarding the pudding ingredients is provided. LCPUFA, long chain polyunsaturated fatty acids; NR, not reported; RCTs, randomised controlled trials.

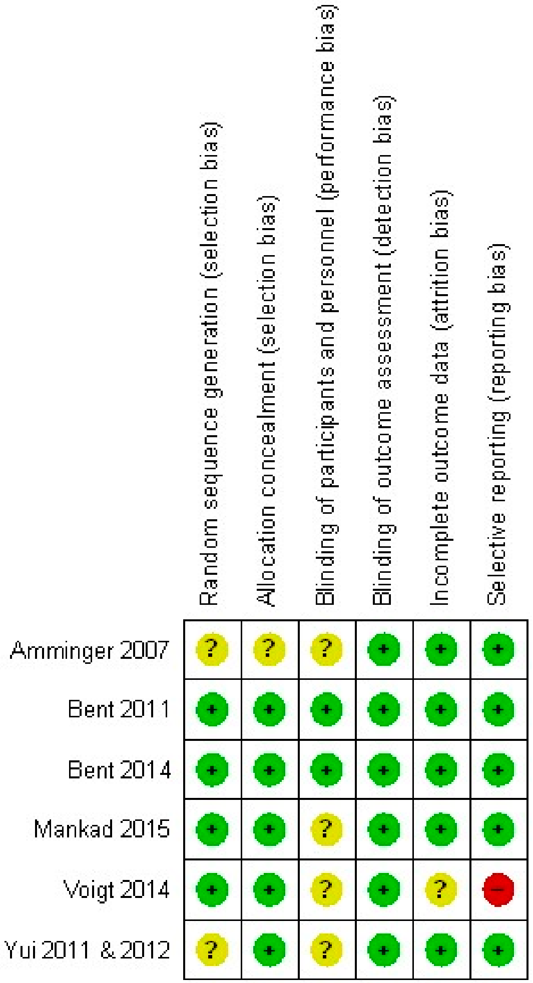

Figure S1. Risk of bias table showing judgments on each risk factor for each primary study included in both meta-analysis and overall interpretation. + = low risk (green); ? = unclear risk (yellow); − = high risk (red.).
